# Mediational Role of Mental Toughness on the Relationship Between Self-Efficacy and Prosocial/Antisocial Behavior in Elite Youth Sport

**DOI:** 10.3389/fpsyg.2021.745323

**Published:** 2021-10-15

**Authors:** Mpaphi Ramolale, Leapetswe Malete, Unhee Ju

**Affiliations:** ^1^Department of Sport Sciences, University of Botswana, Gaborone, Botswana; ^2^Department of Kinesiology, Michigan State University, East Lansing, MI, United States; ^3^Riverside Insights, Itasca, IL, United States

**Keywords:** youth sport, self-efficacy, prosocial behaviors, antisocial behaviors, mental toughness

## Abstract

The modeling and reinforcement of efficacy beliefs and mental toughness in sport continue to generate significant curiosity in the sport psychology research. Investigations into how these behaviors interact and, in the process, affect the development of prosocial and antisocial behaviors among youth athletes are relatively few. This is despite growing evidence of strong associations between self-efficacy beliefs, mental toughness, and various kinds of adaptive and maladaptive behaviors in sport. Therefore, this study sought to examine if mental toughness mediates the relationship between self-efficacy and prosocial/antisocial behaviors in Botswana youth athletes. The study also examined if data from Botswana fit the proposed factor structure of the Sports Mental Toughness Questionnaire, the Self-efficacy Scale, and the Prosocial and Antisocial Behavior in Sport scale. A total of 158 male (*n* = 81) and female (*n* = 77) junior secondary school and senior secondary school (middle and high school) athletes aged 14–20 years old enrolled in Centers for Sport Excellence in Botswana participated in the study. Results showed support for the factor structure of the study's measurement tools. The constancy dimension of mental toughness mediated the relationship between self-efficacy and prosocial/antisocial behavior to teammate and opponent. These findings have implications for research and practice aimed at enhancing efficacy beliefs, mental toughness, and positive youth sport experiences. Contextual relevance of this line of research and measurement tools are discussed.

## Introduction

The role of mental toughness and efficacy beliefs on sport performance has generated significant interest in the sport and exercise psychology literature (Moritz et al., 2000; Chang et al., 2012). The number of studies on these constructs over the last two decades alone, attests to this intrigue (e.g., Stanger et al., 2013; Mazaulan and Abdul Rahim, 2014; Meggs et al., 2014; Habeeb et al., 2019). Also of interest is the growth of research focusing on youth sport. What seems to make these constructs compelling research topics is their ease of application to various achievement contexts in and outside of sport, and how well they can be used to explain other behavior outcomes. While interest in these concepts is largely driven by a desire to explain positive and adaptive behavior outcomes such as confidence, resilience, and drive for success (e.g., Duckworth et al., 2007; Gucciardi et al., 2015; Cormier et al., 2019), their association with maladaptive behaviors such as aggression, cheating, depression, and burnout is equally interesting, but comparatively less explored.

An emerging body of knowledge from mental toughness and the self-efficacy literature suggests significant opportunities exist to develop a better understanding of ways in which these constructs may be associated with prosocial and antisocial behaviors (e.g., Kavussanu and Boardley, 2009; Malete et al., 2013; Hodge and Gucciardi, 2015; Vaughan et al., 2018). For instance, the question of whether maladaptive sense of an individual's capabilities and grit or striving may lead to antisocial behaviors in sport has attracted limited research attention compared the facilitative dimensions of these topic. More investigations along these lines are important to developing a balanced understanding of how mental toughness and efficacy beliefs could be facilitative and debilitative to athletes' success and well-being. This is especially important to strategies aimed at optimizing positive youth sport experiences and personal growth.

The Social Cognitive Theory (SCT: Bandura, 1977, 1986) is a compelling framework for use in this line of work. The SCT proposes that at the core of all the beliefs that people hold about themselves, that affect their day-to-day functioning and standing are self-efficacy beliefs (Bandura, 1986). Self-efficacy is defined as the beliefs that individuals hold about their capabilities to organize and execute a course of action even under challenging circumstances (Bandura, 1997). Therefore, self-referent beliefs are the core argentic factor that determine people's goal directed behavior. The beliefs develop as a consequence of the individual's interactions with the social environment and makes sense of those interactions (Ede et al., 2017).

When used within the context of moral thought and action in sport, the self-efficacy theory would explain why athletes learn good or bad behaviors through observing or receiving reinforcement from significant others such as coaches, opponents, and teammates (Li et al., 2015). Similarly, it would explain why individuals develop moral rules or standards from a variety of sources such as modeling others' evaluative social reactions to antisocial behaviors. In sum, the social environment influences the individual's behavior, but the individual can also affect the environment. The SCT rests on some basic assumptions about learning and behavior. One assumption concerns triadic reciprocity, or the view that personal, behavioral, and environmental factors influence one another in a bidirectional or reciprocal fashion (Bandura, 2001). Another assumption is that people have an agency or ability to influence their own behavior and the environment in a purposeful, goal-directed fashion (Bandura, 2001). This assumption does not deny the importance of the environment in determining behavior, but it argues that people can also, through forethought, self-reflection, and self-regulatory processes, exert substantial influence over their own outcomes and the environment more broadly. The focus of these assumptions on the individual and the environment is why SCT offers a compelling framework for examining the relationship among mental toughness, efficacy beliefs and prosocial/antisocial behaviors in sport. Evidence suggests that, while generally adaptive, mental toughness has a potential maladaptive side to it that may be detrimental to athletic performance as well as health and well-being of athletes (Gucciardi et al., 2015, 2017; Bauman, 2016). Examples of maladaptive behaviors associated with mental toughness are playing with pain and injury, poor decision making, substance abuse, and stigma to seeking help for mental illness (Crust et al., 2014; Bauman, 2016). A likely scenario under which this would happen is when there is a mismatch between an individual's perceived capabilities to execute a behavior or course of action and one sense of mental toughness.

Self-efficacy judgments in the context of mental toughness entail complex processes of self-evaluations of tenancy and persistence under extenuating circumstances (Brace et al., 2020). How these factors relate in their influence on performance and other behaviors in sport has not been widely studied, but the two are understood to have shared attributes (Zeiger and Zeiger, 2018). Being mentally tough means that the athlete has acquired skills in thinking, believing and visualization that enable him/her to effortlessly access empowering emotions during competition. Meggs et al. (2014) examined the proposition that motivation and emotional resiliency in sport stem from differences in core self. They had athletes complete online measures of self-reported mental toughness attribute task. As they predicted, global mental toughness was associated with self-concept positivity, which was particularly high in individuals with positive-integrative self-organization (individuals who distribute positive and negative self-attributes evenly across multiple selves). Specifically, positive integration was associated with constancy (commitment to goal achievement despite obstacles and the potential for failure), which extends presumably from positive integrative emotional stability and drive to resolve negative self-beliefs. These findings have important implications on how efficacy beliefs and mental toughness are likely to interact and affect prosocial and antisocial behaviors in sport.

Other interesting variables to consider alongside the question of how mental toughness and self-efficacy affect adaptive and maladaptive behaviors in sport are, the role of gender, type of sport, and competitive experience. Slimani et al. (2016) examined mental toughness among 677 athletes by type of sport including team sports, individual sports, contact and non-contact sports. Participants were drawn from international, national, county, club/university, and beginner level competitive sports. Results revealed a significant relationship between mental toughness and gender, age, and sporting experience. However, achievement level and the type of sport were not significantly associated with mental toughness. Other findings showed a significant negative relationship between overall mental toughness and sport performance among contact and non-contact sport athletes (Mazaulan and Abdul Rahim, 2014). Therefore, examining these contextual issues is very important to a much broader understanding of how the constructs emerge and may vary between groups and sports contexts.

There has been a significant rise in research on moral reasoning and moral behavior in sport since some of the early work on this topic by Bredemeier and Shields (1986). Several studies have examined dimensions of moral reasoning, prosocial and antisocial behaviors and a wide array of behavior outcomes (Kavussanu et al., 2013; Malete et al., 2013; Hodge and Gucciardi, 2015; Al-Yaaribi and Kavussanu, 2017; Kavussanu and Stanger, 2017). Examples of these behaviors include cheating, aggression, doping, faking an injury, intentionally injuring an opponent to get a competitive advantage, lying to an official, and various forms of gamesmanship. Low reported frequency of engagement in these behaviors has led researchers to infer higher levels of morality or moral reasoning. What is not widely studied but would be of interest is how these behaviors are affected by mental toughness and efficacy beliefs. Research evidence on the reciprocal relationship between sport participation and the development of efficacy beliefs and mental toughness offers a good basis for this line of work. For instance, Carreres-Ponsoda et al. (2017) found that youth participating in out-of-school sport programs have significantly higher levels of self-efficacy, prosocial behavior, personal and social responsibility, and respect, compared to youth participating in other activities or those who reported that they do not take part any kind of activity. These findings offer support to reported benefits of participation in sport widely reported in the positive youth development through sport literature (Gould, 2019; Vella, 2019). The research also offers caution that these benefits are not automatic, and therefore need to be nurtured or intentionally structured into programs.

Research evidence on prosocial and antisocial behaviors suggests that the behaviors depend on myriad factors, including team climate, coaching behaviors, stage of moral reasoning, and personality factors (Hodge and Lonsdale, 2011; Kavussanu et al., 2013; Malete et al., 2013; Fontana et al., 2015). For instance, perceptions of a favorable moral atmosphere and that a coach is effective in instilling an attitude of good moral character in youth athletes has been associated with increased frequency of desirable behaviors even though this does not appear to have any effect on antisocial conduct (Stupuris et al., 2013). Similarly, supportive coach-athlete relationships were associated with both less antisocial and more prosocial behavior in the sports context including attitudes to officials (Šukys and Mankute, 2012). Important covariates identified in the literature are gender, competitive age, and level of experience (Kavussanu et al., 2013). For instance, males, older and more experiences athletes have been found to be more likely to commit more antisocial behaviors or show more moral disengagement than females, younger and less experienced athletes. How these behaviors are associated with efficacy beliefs and mental toughness remains a topic of much curiosity.

It is worth noting that the literature has a lot of spurious findings on many of these relationships. For instance, while participants may report higher prosocial behavior toward teammates and higher antisocial behavior toward opponents (Kavussanu et al., 2013), antisocial behavior to teams mates are not uncommon. Although males are reported to be more likely to commit more antisocial behaviors, Corrion et al. (2009) reported significant effects of affective efficacy on beliefs about cheating in female athletes. Contexts, levels of competition, and the values transmitted by the coaching and team environments are also important considerations (Kavussanu et al., 2013; Malete et al., 2013). At the level of an individual, the effects can also be mediated by moral reasoning and ego orientation, suggesting a bracketed morality concept to prosocial and antisocial behavior. Therefore, individual factors, as well as contextual/cultural differences on perceived purposes and value of sport and how children and youth get socialized into sport need to be considered when examining the relationships. Overall, it seems self-efficacy affects self-regulatory mechanisms governing the acceptability and likelihood of prosocial and antisocial behaviors in sports.

### The Present Study

The purpose of this study was to examine if mental toughness mediates the relationship between self-efficacy and prosocial and antisocial behavior in elite youth sport. The study sough to answer the following research questions:

1. Does data from Botswana youth athletes fit the proposed factor structure of the Sports Mental Toughness Questionnaire (SMTQ), General Self-efficacy Scale (GSES), and the Prosocial and Antisocial Behavior in Sport Scale (PABS)?
2. Does mental toughness mediate the relationship between self-efficacy and prosocial and antisocial behavior in elite youth sport?

Based on these research questions we tested the following hypotheses:

1. Data from elite youth athletes in Botswana fit the existing factor structure of the SMTQ GSES, and the PABS.
2. Mental toughness mediates the relationship between self-efficacy and prosocial and antisocial behavior in elite youth sport.

This study hopes to add to the extant literature on efficacy beliefs, mental toughness, prosocial and antisocial behaviors in elite youth sport, and in particular address existing evidence gaps on how mental toughness could mediate the relationship between efficacy beliefs and various behaviors in sport. Our choice of Botswana elite youth sport for this study offers an opportunity to examine this relationship in a context where sport is less commercialized and relies largely on public funding compared to contexts where most of the research on this topic has been conducted. A premise that context may affect how constructs such as mental toughness develop and are viewed makes the study interesting. Earlier research on coaching efficacy and athlete level moral variables, youth aggression and antisocial behaviors in Botswana offer a basis for examining other dimensions of this line of research (Malete et al., 2013).

## Materials and Methods

### Participants

Participants for this study were 158 junior secondary school and senior secondary school athletes enrolled in Centers for Sport Excellence in Botswana with 14–20 years of age (51% male, 49% female; 83% team sport, 17% individual sport; see Table 1 for further demographic details).

**Table 1:** Demographics (*N* = 158).

**Variable**	***n***	**Percent**
Gender		
Male	81	51.3
Female	77	48.7
Major sport		
Football	41	25.9
Netball	16	10.1
Athletics (Track and Field)	21	13.3
Volleyball	38	24.1
Softball	36	22.8
Boxing	6	3.8
Extracurricular activity		
Yes	78	49.4
No	80	50.6
Sport type		
Team sport	131	82.9
Individual sport	27	17.1

### Procedures

Ethical clearance was obtained from the Institutional Review Boards of the University of Botswana, the Ministry of Youth, Sports and Culture, and the Ministry of Education and Skills Development. The first author traveled to each of the six Centers for Sport Excellence around the country (South, South East, North, Central and North East) to recruit participants and administer the questionnaires. The data collection process was done over two days. On the first day, the researcher held short meetings with center managers and athletes to introduce the study and recruit participants. Youth athletes who expressed interest in taking part in the study were given parent consent and youth assent forms to take home for signing. They were asked to return the signed forms the next day. The forms had an introductory message about study which also stated the rights of participants and their parents to decline participation or to answer any questions. On the second day, with the help of team managers, athletes were met at the dining hall after school to complete the survey. Only participants that brought back signed parental consent forms were allowed to complete the survey. Out of target population of 256 youth athletes at the Centers for Sport Excellence, 165 returned forms and expressed interest to participate in the study. Seven were excluded because they did not have signed parental consent forms. This resulted in 158 completed surveys, representing 61.7% response rate. None of the respondents who started the survey withdrew from the study. Each questionnaire administration session lasted ~60 min.

### Measures

#### Background Information

Participants completed a background information questionnaire that collected information on age, gender, type of sport, and extra-curricular activities the youth typically engaged in, as well as the frequency of participation in sport.

#### Mental Toughness

The SMTQ (Sheard et al., 2009) was used to assess participants' mental toughness. The SMTQ is a 14-item questionnaire with items anchored on a 5*-*point Likert scale from 1 (*strongly disagree*) to 5 (*Strongly disagree*). It has three sub-scores: (1) *Confidence* (e.g., “I have an unshakable confidence in my ability”); (2) *Constancy* (e.g., “I am committed to complete the task I have to do”); and (3) *Control* (e.g., “I am overcome by self-doubt”). The STMQ has been reported to possess high internal consistency with Cronbach's alphas >0.70 for all three subscales (Sheard et al., 2009).

#### Prosocial and Antisocial Behavior

Prosocial and antisocial behavior in sport was measured using the PABS (Kavussanu and Boardley, 2009). The PABS has 20 items anchored on a 5*-*point Likert scale. Participants were asked to state how often they engaged in prosocial and antisocial behaviors on a scale of 1 (*never*) to 5 (*very often*). A typical item for prosocial behaviors is, “Helped an opponent off the floor” while an example of an antisocial behavior would be, “Deliberately fouled an opponent.” The PABS consists of four sub-scores: (1) *antisocial* opponent, (2) *antisocial* teammate, (3) *prosocial* opponent, and (4) *prosocial* teammate. The initial development of the PABS reported support for the scale's discriminant and concurrent validity as well as use in team sport (Kavussanu and Boardley, 2009).

#### Self-Efficacy

Participants' self-efficacy was measured using the GSES (Schwarzer and Jerusalem, 1995). The GSES has 10 items measured on a 4-point Likert scale. A typical item is, “I am confident that I could deal efficiently with unexpected events.” Participants rate themselves on a scale of 1 (*not at all true*) to 4 (*exactly true*) and can obtain scores ranging from 10–40. The GSES has been reported to have reliability, stability and construct validity across contexts and cultures using different languages with Cronbach's alphas ranging between 0.86 and 0.94 (Schwarzer and Jerusalem, 1995).

### Data Analysis

We primarily conducted path analysis using M*plus* 7.4 (Muthén and Muthén, 2009). To address non-normality in the measures for self-efficacy, mental toughness, and prosocial and antisocial behavior, a robust maximum likelihood estimator (MLR) was used to fit models with the correction of standard errors. Since we do not have any missing values in the measures of data, further approaches to handle missing data were not needed.

Based on the factor structures of each of three constructs confirmed using confirmatory factor analysis (CFA) models, we created manifest variables representing the (sub) scale scores for the three constructs by simply averaging the item responses associated with the scales. We then conducted a path analysis using the manifest indicators to examine the mediation effects of mental toughness on the relation between self-efficacy and prosocial and antisocial behavior. This would make a model more parsimonious with quite small sample size (*N* = 158) but many variables. Different mediator variables (age, gender, sport-type, and extra-curricular activities) were included in preliminary model testing and only gender was statistically significant. Therefore, a decision was made to only include gender as a covariate in model testing. The path analysis models considered in the study were evaluated using the following fit statistics: chi-square statistics, the comparative fit index (CFI) and the Tucker-Lewis index (TLI), the root mean square error of approximation (RMSEA) and the standardized root mean square residuals (SRMR). TLI and CFI values >0.90 were considered as acceptable, and RMSEA and SRMR values below 0.08 were acceptable (e.g., Hu and Bentler, 1999; Kline, 2005).

## Results

### Preliminary Confirmatory Factor Analysis

To confirm the three-factor structure of mental toughness, we conducted a CFA using the 14 items with the two items (Item 7, Item 8) reversely coded. Results showed that the model did not fit the data well, as χ^2^(74) = 120.74, CFI = 0.76, TLI = 0.70, and RMSEA = 0.06. Thus, we removed two items (Item 1, Item 9) with the standardized factor loadings lower than 0.30 from the scale, resulting in the improvement of model fit, χ^2^(51) = 64.24, CFI = 0.92, TLI = 0.89, and RMSEA = 0.04, with high factor loadings, which is a final model for the structure of mental toughness. Also, we noticed that the scale appeared to violate the assumptions of Cronbach α (e.g., tau-equivalent model, unidimensionality), causing the underestimation of reliability. Hence, the composite reliability, or coefficient omega (ω; McDonald, 1999), was computed based on the factor model with maximum likelihood estimator (Raykov and Marcoulides, 2012). The composite reliability was ω = 0.60 with 95% CI [0.50, 0.70].

We evaluated the four-factor structure of prosocial and antisocial behavior in the data using a CFA. As a result, fit of the model to the data was acceptable with moderate to high factor loadings, as χ^2^(164) = 203.60, CFI = 0.90, TLI = 0.87, and RMSEA = 0.04. Based on the factor model, we computed the composite reliability, which was ω = 0.70 with 95% CI [0.62, 0.78].

We tested a single-factor model of the GSES to measure self-efficacy using a CFA. Although the fit statistics of the model was good [χ^2^(35) = 37.30, CFI = 0.97, TLI = 0.97, and RMSEA = 0.02], the factor loadings of two items (Item 7, Item 8) were about 0.20. For the revision of the scale, we sequentially removed each of the two items and checked the pattern of the loadings. The final single-factor model with eight items showed the perfect model fit statistics [χ^2^(20) = 18.11, CFI/TLI = 1.00, and RMSEA = 0.00] and high factor loadings of all the items. In this case, as the GSES was satisfied with assumptions of Cronbach' α (e.g., unidimensionality, tau-equivalent model uncorrelated error; see Raykov and Marcoulides, 2011; Raykov et al., 2017), we computed both coefficient alpha and coefficient omega. As expected, the two reliability values were identical, which was 0.65 with 95% CI [0.55, 0.75].

### Descriptive Statistics and Correlations

Table 2 displays means and standard deviations of the manifest measures for constructs of interest, as well as their correlation coefficients. Among the subscales of SMTQ, participants had the highest average scores on constancy (*M* = 4.03, *SD* = 0.78) while the lowest on control (*M* = 3.03, *SD* = 0.90). Antisocial behavior showed on average less frequency, whereas prosocial behavior reported high frequency. We also evaluated univariate normality of the measures considered, reporting normality for each of the SMTQ factors but deviations from normality for prosocial and antisocial behavior as well as self-efficacy. Multivariate normality was not satisfied (Mardia skewness = 9.47, Mardia kurtosis = 86.67, *p* < 0.001). To address the non-normality issue, the models were estimated with the MLR estimator available in M*plus* 7.4 (Muthen and Satorra, 1995).

**Table 2:** Descriptive statistics and correlation coefficients among variables (*N* = 158).

**Variables**	**Mental toughness**			**Prosocial/Antisocial behavior**				**Self-efficacy**
	**1**	**2**	**3**	**4**	**5**	**6**	**7**	**8**
1. Control	1.00							
2. Constancy	0.26^*^	1.00						
3. Confidence	−0.05	−0.45^*^	1.00					
4. Antisocial opponent	0.09	−0.31^*^	−0.15	1.00				
5. Antisocial teammate	0.14	−0.40^*^	−0.20^*^	0.46^*^	1.00			
6. Prosocial opponent	−0.07	0.17^*^	0.05	0.14	0.03	1.00		
7. Prosocial teammate	0.09	0.36^*^	0.31^*^	−0.15	−0.20^*^	0.22^*^	1.00	
8. Self-efficacy	−0.01	0.36^*^	0.27^*^	−0.18^*^	−0.23^*^	0.14	0.27^*^	1.00
*M*	3.03	4.03	3.58	1.60	1.60	2.67	3.08	3.03
*SD*	0.90	0.78	0.73	0.49	0.48	0.84	0.65	0.54
Min.	1.00	1.75	1.80	1.00	1.00	1.00	1.25	1.13
Max.	5.00	5.00	5.00	3.00	3.20	4.00	4.00	4.00
Skewness	−0.18	−0.66	−0.13	0.79	0.88	−0.20	−0.60	−0.71
Kurtosis	−0.27	−0.30	−0.61	−0.18	0.32	−0.91	−0.08	0.71

* *p <0.05*.

Regarding a pattern of correlations, constancy was negatively correlated with confidence (*r* = −0.45) but positively correlated with control (*r* = 0.26). Constancy had negative relationships with antisocial opponent (*r* = −0.31) and antisocial teammate (*r* = −0.40) but had low to moderate positive correlations with prosocial opponent (*r* = 0.17) and prosocial teammate (*r* = 0.36). Confidence was negatively correlated with antisocial teammate (*r* = −0.20) but positively correlated with prosocial teammate (*r* = 0.31). Control had no relationships with any facets of prosocial and antisocial behavior. In addition, self-efficacy was significantly correlated with confidence (*r* = 0.27) and constancy (*r* = 0.36) as well as prosocial and antisocial behavior (−0.23 < *r*s <0.27) but not with control (*r* = −0.01, *p* = 0.91) and proposal opponent (*r* = 0.14, *p* = 0.09).

### Path Analysis

**Figure 1:**
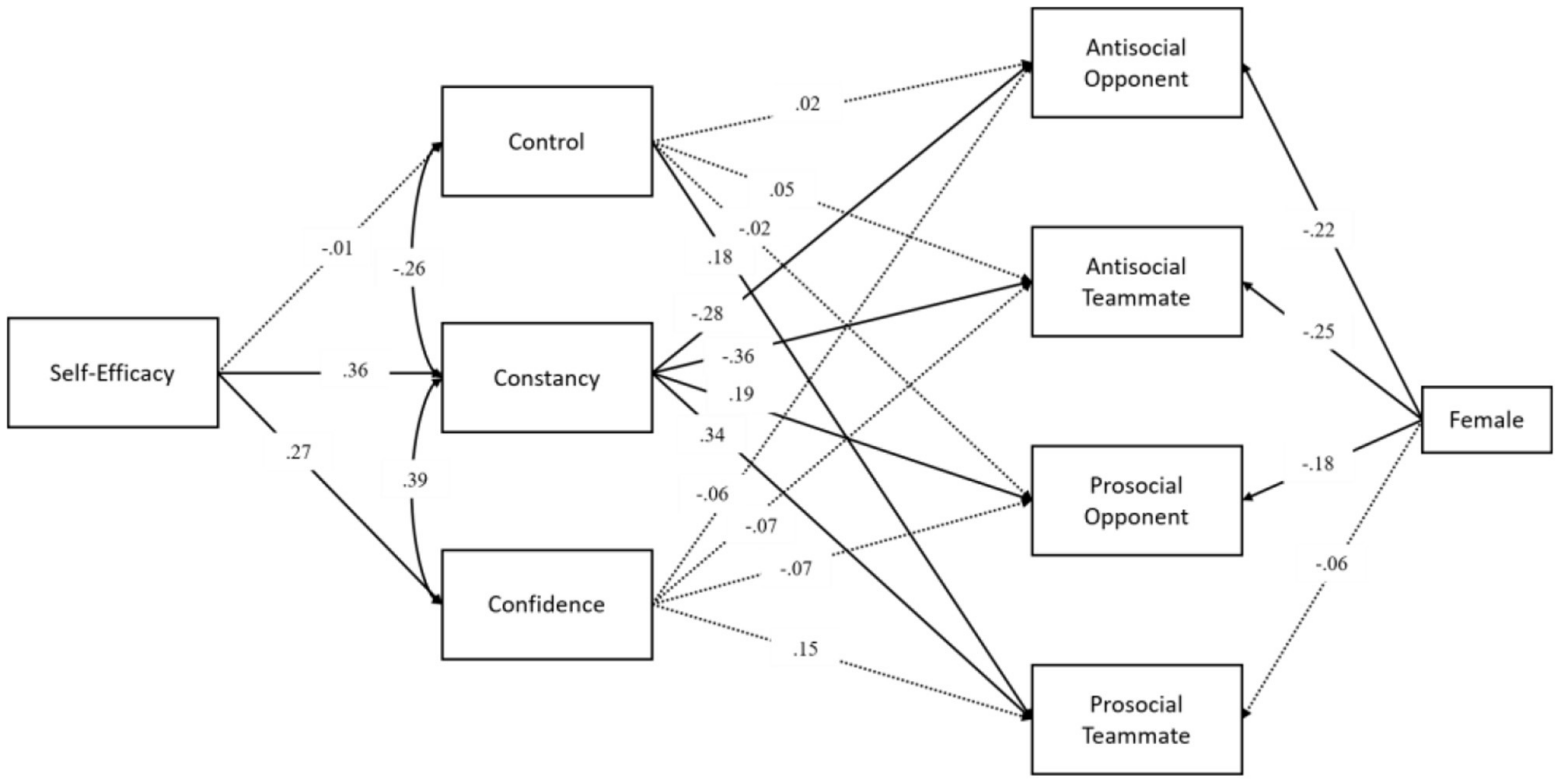
Path diagram and standardized path coefficients for the relationship among self-efficacy, mental toughness, and prosocial and antisocial behavior. Female indicates the dummy coding of gender.

The relationship among self-efficacy, mental toughness, and prosocial and antisocial behavior was tested using the path analysis model in Figure 1. Fit of the model was excellent to the data, as χ^2^(8) = 10.20 with *p* = 0.25, CFI = 0.99, TLI = 0.94, RMSEA = 0.04, and SRMR = 0.04. As an expected pattern of correlations, self-efficacy was significantly associated with constancy (*B* = 0.51, *SE* = 0.12, *p* < 0.001) and confidence (*B* = 0.36, *SE* = 0.11, *p* = 0.001), but not with control (*B* = −0.01, *SE* = 0.13, *p* = 0.92).

Regarding the relation between mental toughness and prosocial/antisocial behavior, only constancy significantly predicted prosocial and antisocial behavior, after accounting for the significant contributions of self-efficacy on mental toughness as well as gender on prosocial and antisocial behavior. It is worth noting that for the effect of gender as a covariate, males reported significantly higher prosocial/antisocial opponent and antisocial teammate than females (−0.22 < *B*s < −0.30, 0.07 < *SE*s <0.13, 0.001 < *p*s <0.03) with small effect size (−0.18 < βs < −0.25). Constancy was negatively associated with antisocial behavior (*B* = −0.18, *SE* = 0.05, *p* = 0.001 for opponent; *B* = −0.22, *SE* = 0.05, *p* = 0.001 for teammate), whereas positively related with prosocial behavior (*B* = 0.21, *SE* = 0.10, *p* = 0.04 for opponent; *B* = 0.28, *SE* = 0.08, *p* = 0.001 for teammate). Based on the effect size using the standardized coefficients, constancy did more strongly predict antisocial (β = −0.36) and prosocial teammate (β = 0.34) than the opponent.

More importantly, the results for the indirect effects of mental toughness indicated that constancy fully mediated the relationship of self-efficacy to antisocial and prosocial behavior, particularly for antisocial opponent (*B* = −0.09, *SE* = 0.04, β = −0.10, *p* = 0.01), for antisocial teammate (*B* = −0.11, *SE* = 0.04, β = −0.13, *p* = 0.003), and for prosocial teammate (*B* = 0.14, *SE* = 0.05, β = *0.1*2, *p* = 0.01) but not for prosocial opponent (*B* = 0.11, *SE* = 0.06, β = *0.0*7, *p* = 0.07), controlling for gender. However, other mental toughness factors (i.e., control and confidence) did not significantly mediate the relationship between self-efficacy and prosocial/antisocial behavior.

## Discussion

Evidence suggests that sport provides an opportunity for young people to learn and develop a range of positive psychosocial and behavioral outcomes (Weiss, 2013). However, the competitive nature of sport can potentially result in learning and engaging in negative social behaviors that can have adverse effects on their own development and that of others (Bredemeier and Shields, 1986). Given that sport does not automatically build moral character, identifying how self-efficacy and mental toughness may affect the development of prosocial and antisocial behaviors in young athletes is very important. Further, examining the strength and contextual relevance of existing measurement tools is especially critical to continued efforts to studying these behaviors because broader application of the tools obviates the need to develop new ones for each context and population. Therefore, we sought to investigate if mental toughness mediates the relationship between self-efficacy and prosocial and antisocial behaviors of Botswana youth athletes. In the process we examined if data from Botswana would support the factor structure of self-efficacy, mental toughness, and prosocial and antisocial behavior scales selected for this study.

First, our data from Botswana confirmed the factor structures of the GSES (Schwarzer and Jerusalem, 1995), the SMTQ (Sheard et al., 2009) and the PABS (Kavussanu and Boardley, 2009) used to measure self-efficacy, mental toughness, and prosocial and antisocial behavior, respectively. Although two items had to be excluded from the original SMTQ and one from the PABS during confirmatory factor analysis, overall, the results provide evidence that support the construct validity and reliability of these measures with data from Botswana. This suggests these tools are relevant for use in a Botswana youth sport context with a potential broader application in contexts where they were not developed and normed.

Our findings on the psychometric properties and suitability of the SMTQ in Botswana are particularly interesting given a previous study that showed divergence of the item-factor loadings from the original scale when used with South African competitive tennis players (Cowden and Meyer-Weitz, 2016). Cowden and Meyer-Weitz (2016) explained this divergence might have been due to race and cultural diversity of their sample, the sport context, and the fact that they only focused on one sport (tennis), whereas the original scale was developed using data drawn from participants in diverse sports. These issues and the fact that participants in the Cowden and Meyer-Weitz (2016) study were older, are important to consider when evaluating different findings in studies of measurement fit across contexts. Even with these differences, our findings on the factor structure of the SMTQ are consistent with what was reported by Cowden and Meyer-Weitz (2016). Regarding the GSES and the PABS, to our knowledge, none of previous studies have used these instruments in the similar African context. However, the strength of the instruments' psychometric properties with a Botswana sample gives us confidence in their future use in research involving youth athletes from similar sports contexts. The strength of the GSES in this study is in line with what was reported in previous multicultural validation studies, even though none involved African populations (Luszczynska et al., 2005).

The pattern of correlations among study variables were plausible and not out of the ordinary. For instance, self-efficacy had a moderate, positive correlation with prosocial behavior to teammates but a negative correlation with antisocial behaviors to teammates and opponents. Though not a direct comparison, this is consistent with Kavussanu and Boardley's (Kavussanu and Boardley, 2009) findings that participants were likely to show prosocial behavior to teammates and antisocial behavior to opponents. The moderate, positive correlation between self-efficacy and the constancy and confidence dimensions of mental toughness is consistent with expected relationships between efficacy beliefs and sport confidence reported in the literature (Vealey and Knight, 2002; Vealey and Chase, 2008). The inter-correlations among the three mental toughness factors did not fully support their positive relations reported by Sheard et al.'s (Sheard et al., 2009), initial study of development and validation of the mental toughness questionnaire, but they were also not out of the ordinary. The moderate, positive correlation between confidence and constancy is to be expected given that the two factors entail similar characteristics, such as bouncy and goal directed behavior.

Regarding the hypothesized mediational role of mental toughness in the relationship between self-efficacy and prosocial and antisocial behavior, only constancy was significantly associated with prosocial and antisocial behaviors, after accounting for the contributions of self-efficacy on mental toughness as well as gender on prosocial and antisocial behavior. Constancy was negatively associated with antisocial behavior but positively related with prosocial behavior. Our findings suggest that the higher participants rated themselves on adaptive behaviors like commitment to task completion, setting challenging task, and persistence, the more likely they were to show higher prosocial behavior and low antisocial behavior to teammates and opponents. This is consistent with what has been previously reported in the literature looking at similar behaviors. For instance, Meggs et al. (2014) found significant positive associations between constancy and positive integration. Elements of positive self-concept that they mentioned include emotional stability and drive to resolve negative beliefs. Similar to Meggs et al.'s (Meggs et al., 2014) arguments that athlete capabilities and optimism may mitigate feelings of frustration and tension, it seems the sense of constancy (related to commitment to task completion, setting challenging task, and persistence) among youth athletes in our study reduces their likelihood to show antisocial behaviors while the opposite may be true. This suggests a capacity to compartmentalize goal directed behavior, similar to what Kavussanu and Boardley (2009) described as bracketed morality in their study of prosocial and antisocial behavior to teammates and opponents.

We found that the *teammate* facet of prosocial/antisocial behavior is more strongly related to mental toughness (i.e., constancy) than the *opponent* dimension. This is in line with previous findings that youth athletes are likely to display more antisocial behaviors toward their opponents than their teammates and more prosocial behavior toward their teammates than toward their opponents (Bruner et al., 2014; Kavussanu and Stanger, 2017). Similar to previous findings, our findings suggest that prosocial/antisocial behavior is likely to be mitigated by group membership and feelings associated with the group (Bruner et al., 2014) as well as goal directed behavior. Compartmentalization of moral reasoning might be in play here as well (Meggs et al., 2014; Showers et al., 2015).

It is important to note the effect of gender as a covariate, where males reported significantly higher antisocial behavior to opponent and teammate and higher prosocial behavior to opponent than females. This is consistent with previous studies that found that males tend to score higher on measures of antisocial behavior than females (Hodge and Lonsdale, 2011; Bruner et al., 2014). That said, it is worth noting that gender differences on antisocial/prosocial behavior remain highly inconsistent, most likely due to sport context and sample characteristics.

It is also important to discuss lack of significant effects for other covariates (age, sport type and participation in extra-curricular activities). Contrary to previous studies that reported linear age differences in prosocial and antisocial behaviors (Bredemeier, 1995; Kavussanu et al., 2006; Malete et al., 2013) no significant differences were found in the current study. This is surprising considering the trend on age related differences and the fact that a previous study in the same context with a similar age group reported age related differences in prosocial and antisocial behaviors (Malete et al., 2013). However, age related differences can be tenuous because of confounding sport context factors such as motivational climate, moral atmosphere, team norms and the role of coaches. These have consistently been associated with prosocial and antisocial behaviors in the literature (Rutten et al., 2011; Malete et al., 2013; Kavussanu and Stanger, 2017). Similar to age, effects for type of sport, specifically individual vs. team sports were not significant in the current study. This is in contrast to findings reported by Rutten et al. (2011), where youth athletes in individual sports like track and field reported less antisocial behavior than athletes involved in team sports like soccer. The lack of significant effects in the current study suggests that the way individual and teams sports are organized in this context may limit differences in prosocial and antisocial behaviors. However, caution is advised in the interpretation of this finding because of a smaller sample size for individual sports compared to team sports that could have had an effect on these findings. The effects of participation in extra-curricular activities on the path model was considered relevant because of the potential role of the activities in nurturing a collegial team or sport environment that could influence the outcome variables. These effects were also not significant. While there is limited research and conceptual frameworks from which to draw comparisons on this relationship, this result may have a lot do with the frequency of participation than the quality and effectiveness of the programs the youth participated in. About 75% of the youth in this study indicated that they spent ~5 days a week practicing or playing their sport. This means they had limited time to do extra-curricular activities. Overall, the lack of significant effects for all these covariates may be due to the fact that elite youth athletes tend to self-select and have very little to differentiate between them.

### Limitations and Suggestions for Future Research

Although our study has many strengths, there are a number of limitations that warrant consideration. First, the cross-sectional design used in this study limits the ability to infer causal relationship among self-efficacy, mental toughness, and prosocial/antisocial behavior of youth athletes in the study. Although the sample size was large enough to enable us to investigate the hypothesized relationships, a larger sample of youth athletes is likely to have increased statitical power and strengthened the external validity of our findings. Because of this, caution still has to be excercised when generalizing findings to broader youth athlete population. However, the high response rate from the taget population gives us confidence that we had a representative sample of youth in this elite group. Another limitation to the study is that we did not control for social desirability of responses to prosocial/antisocial behevavior items, given that they tend to be susceptible to social desirability. Therefore we cannot rule out response bias to items on antisocial behaviors. Future research could benefit from controling for social desirability and recruiting larger samples of diverse youth athletes to address this. Use of mixed methods, where additional focus groups are run with a sub-sample of participants, is likey to strengthen the results. It is likely that participants had challenges with their interpretation of control-related items of the SMTQ. Future research measuring mental toughness and prosocial/antisocial behavior could benefit from using other approaches that make these behaviors more vivid such as presenting hypothetical scenarios and asking participants to evaluate how they would respond to those behaviors. Success with this approach has been demonstrated in previous research (Malete et al., 2013).

## Conclusions

Findings from our study offer valuable insights on the measurement strength and relevance of the test tools we used in this study in another context. Specifically the findings support the factor structure of the GSES, the SMTQ, and the PABS with a Botswana sample. The findings also demonstrate that mental toughness does mediate the relationship between self-efficacy and prosocial/antisocial behavior in elite youth athletes. Specifically this relationship was accounted for by the constancy dimension of mental toughness. The higher participants rated themselves on adaptive behaviors like commitment to task completion, setting challenging task and persistence, the more likely they were to show higher prosocial behavior and low antisocial behavior to teammates and opponents. Overall, these findings suggests more reseach is needed that looks into the relationship among self-efficacy, mental toughness and prosocial and antisocial behaviors in youth sport. Future research could use multidimensional self-efficacy scales and specific prosocial behaviors like athlete altruistic behaviors to teammates and opponents during play and common antisocial behaviors like gamesmanship. There is also need to use more robust designs to collect nuanced data on the effects of the covariates in these relationships.

## Data Availability Statement

The original contributions presented in the study are included in the article/Supplementary Material, further inquiries can be directed to the corresponding author/s.

## Ethics Statement

The studies involving human participants were reviewed and approved by University of Botswana and Ministry of Youth, Sport and Culture, Botswana. Written informed consent to participate in this study was provided by the participants' legal guardian/next of kin.

## Author Contributions

MR and LM contributed to the conception and design of the study and wrote several sections of the manuscript. UJ conducted the statistical analyses and wrote the results section. All authors contributed to the manuscript revision and read and approved the submitted version of the manuscript.

## Conflict of Interest

UJ was employed by the Riverside Insights. The remaining authors declare that the research was conducted in the absence of any commercial or financial relationships that could be construed as a potential conflict of interest.

## Publisher's Note

All claims expressed in this article are solely those of the authors and do not necessarily represent those of their affiliated organizations, or those of the publisher, the editors and the reviewers. Any product that may be evaluated in this article, or claim that may be made by its manufacturer, is not guaranteed or endorsed by the publisher.

## References

[B1] Al-Yaaribi A. Kavussanu M. (2017). Teammate prosocial and antisocial behaviors predict task cohesion and burnout: the mediating role of affect. J. Sport Exerc. Psychol. 39, 199–208. 10.1123/jsep.2016-0336 28891748

[B2] Bandura A. (1977). Self-efficacy: toward a unifying theory of behavioral change. Psychol. Rev. 84:191. 10.1037/0033-295X.84.2.191 847061

[B3] Bandura A. (1986). Social Foundations of Thought and Action: A Social Cognitive Theory. Hoboken, NJ: Prentice-Hall.

[B4] Bandura A. (1997). Self-efficacy: The Exercise of Control. New York, NY: Freeman.

[B5] Bandura A. (2001). Social cognitive theory: an agentic perspective. Annu. Rev. Psychol. 52, 1–26. 10.1146/annurev.psych.52.1.1 11148297

[B6] Bauman N. J. (2016). The stigma of mental health in athletes: are mental toughness and mental health seen as contradictory in elite sport? Br. J. Sports Med. 50, 135–136. 10.1136/bjsports-2015-095570 26626270

[B7] Brace A. W. George K. Lovell G. P. (2020). Mental toughness and self-efficacy of elite ultra-marathon runners. PLoS ONE 15, e0241284–e0241284. 10.1371/journal.pone.0241284 33147236 PMC7641431

[B8] Bredemeier B. J. Shields D. L. (1986). Athletic aggression: an issue of contextual morality. Soc. Sport J. 3:15. 10.1123/ssj.3.1.15

[B9] Bredemeier B. J. L. (1995). Divergence in children's moral reasoning about issues in daily life and sport specific contexts. Int. J. Sport Psychol. 26, 453–463.

[B10] Bruner M. W. Boardley I. D. Côté J. (2014). Social identity and prosocial and antisoclai behavior in youth sport. Psychol. Sport Exerc. 15, 56–64. 10.1016/j.psychsport.2013.09.00329058105

[B11] Carreres-Ponsoda F. Escart,í A. Llopis-Goig R. Cortell-Tormo J. M. (2017). The effect of an out-of-school mindfulness program on adolescents' stress reduction and emotional wellbeing. Cuadernos De Psicología Del Deporte 17, 35–44.

[B12] Chang Y. Chi L. Huang C. (2012), Mental toughness in sport: a review. Int. J. Sport Exerc. Psychol. 10, 79–92. 10.1080/1612197X.2012.661202

[B13] Cormier D. L. Dunn J. G. H. Dunn J. C. (2019). Examining the domain specificity of grit. Pers. Individ. Dif. 139, 349–354. 10.1016/j.paid.2018.11.026

[B14] Corrion K. Long T. Smith A. L. d'Arripe- Longueville F. (2009). “It's not my fault: It's not serious”: athletes accounts of moral disengagement in competitive sport. Sport Psychol. 23, 388–404. 10.1123/tsp.23.3.388

[B15] Cowden R. G. Meyer-Weitz A. (2016). Self-reflection and self-insight predict resilience and stress in competitive tennis. Soc. Behav. Pers. 44, 1133–1149. 10.2224/sbp.2016.44.7.1133

[B16] Crust L. Earle K. Perry J. Earle F. Clough A. Clough P. (2014). Mental toughness in higher education: relationships with achievement and progression in first-year university sports students. Personal. Individ. Diff. 69, 87–91. 10.1016/j.paid.2014.05.016

[B17] Duckworth A. L. Peterson C. Matthews M. D. Kelly D. R. (2007). Grit: perseverance and passion for long-term goals. J. Pers. Soc. Psychol. 92, 1087–1101. 10.1037/0022-3514.92.6.1087 17547490

[B18] Ede A. Sullivan P. J. Feltz D. L. (2017). Self-doubt: uncertainty as a motivating factor on effort in an exercise endurance task. Psychol. Sport Exerc. 28, 31–36. 10.1016/j.psychsport.2016.10.002

[B19] Fontana M. Bass J. R. Fry M. D. (2015). From Smith center to Coney Island: examining the coaching climate in the united states sporting culture. J. Contemp. Athl. 9:211.

[B20] Gould D. (2019). The current youth sport landscape: identifying critical research issues. Kinesiol. Rev. 8, 150–161. 10.1123/kr.2019-0034

[B21] Gucciardi D. F. Hanton S. Fleming S. (2017). Are mental toughness and mental health contradictory concepts in elite sport? A narrative review of theory and evidence. J. Sci. Med. Sport 20, 307–311. 10.1016/j.jsams.2016.08.006 27568074

[B22] Gucciardi D. F. Hanton S. Gordon S. Mallett C. Temby P. (2015). The concept of mental toughness: tests of dimensionality, nomological network, and traitness. J. Pers. 83, 26–44. 10.1111/jopy.12079 24428736

[B23] Habeeb C. M. Eklund R. C. Coffee P. (2019). Reciprocal relationships between efficacy and performance in athlete dyads: self-, other-, and collective constructs. J. Sport Exerc. Psychol. 41, 147–158. 10.1123/jsep.2018-0248 31170872

[B24] Hodge K. Gucciardi D. F. (2015). Antisocial and prosocial behavior in sport: the role of motivational climate, basic psychological needs, and moral disengagement. J. Sport Exerc. Psychol. 37:257. 10.1123/jsep.2014-0225 26265339

[B25] Hodge K. Lonsdale C. (2011). Prosocial and antisocial behavior in sport: the role of coaching style, autonomous vs. controlled motivation, and moral disengagement. J. Sport Exerc. Psychol. 33, 527–547. 10.1123/jsep.33.4.527 21808078

[B26] Hu L. Bentler P. M. (1999). Cutoff criteria for fit indexes in covariance structure analysis: conventional criteria versus new alternatives. Struct. Equ. Model. 6, 1–55. 10.1080/10705519909540118

[B27] Kavussanu M. Boardley I. D. (2009). The prosocial and antisocial behavior in sport scale. J. Sport Exerc. Psychol. 31, 97–117. 10.1123/jsep.31.1.97 19325190

[B28] Kavussanu M. Seal A. Phillips D. (2006). Observed prosocial and antisocial behaviors in male soccer teams: age differences across adolescence and the role of motivational variables. J. Appl. Soc. Psychol. 18, 326–344. 10.1080/10413200600944108

[B29] Kavussanu M. Stanger N. (2017). Moral behavior in sport. Curr. Opin. Psychol. 16, 185–192. 10.1016/j.copsyc.2017.05.010 28813348

[B30] Kavussanu M. Stanger N. Boardley I. D. (2013). The prosocial and antisocial behaviour in sport scale: further evidence for construct validity and reliability. J. Sports Sci. 31, 1208–1221. 10.1080/02640414.2013.775473 23472827

[B31] Kline T. (2005). Psychological Testing: A Practical Approach to Design and Evaluation. New York, NY: Sage. 10.4135/9781483385693

[B32] Li C. Koh K. T. Wang C. K. J. Chian L. K. (2015). Sports participation and moral development outcomes: examination of validity and reliability of the prosocial and antisocial behavior in sport scale. Int. J. Sports Sci. Coach. 10, 505–513. 10.1260/1747-9541.10.2-3.505

[B33] Luszczynska A. Scholz U. Schwarzer R. (2005). The general self-efficacy scale: multicultural validation studies. J. Psychol. 139, 439–457. 10.3200/JRLP.139.5.439-457 16285214

[B34] Malete L. Chow G. M. Feltz D. L. (2013). Influence of coaching efficacy and coaching competency on athlete-level moral variables in Botswana youth soccer: coaching efficacy and competency. J. Appl. Soc. Psychol. 43, 2107–2119. 10.1111/jasp.12164

[B35] Mazaulan M. Abdul Rahim M. R. (2014). “Relationship between mental toughness and sport performance among contact and non-contact sport athletes,” in Proceedings of the International Colloquium on Sports Science, Exercise, Engineering and Technology 2014 (ICoSSEET 2014), eds R. Adnan, S. Ismail, N. Sulaiman (Singapore: Springer), 411–419. 10.1007/978-981-287-107-7_43

[B36] McDonald R. P. (1999). Test Theory: A Unified Treatment. Mahwah, NJ: Lawrence Erlbaum.

[B37] Meggs J. Ditzfeld C. Golby J. (2014). Self-concept organisation and mental toughness in sport. J. Sports Sci. 32, 101–109. 10.1080/02640414.2013.812230 23968218

[B38] Moritz S. E. Feltz D. L. Fahrbach K. R. and. Mack D. E. (2000). The relation of self-efficacy measures to sport performance: a meta-analytic review. J. Sport Exerc. Psychol. 71, 280–294. 10.1080/02701367.2000.10608908 10999265

[B39] Muthén B. Muthén B. O. (2009). Statistical Analysis with Latent Variables. Hoboken, NJ: Wiley.

[B40] Muthen B. O. Satorra A. (1995). complex sample data in structural equation modeling. Sociol. Methodol. 25, 267–316. 10.2307/271070

[B41] Raykov T. Marcoulides G. A. (2011). Introduction to Psychometric Theory. New York, NY: Routledge. 10.4324/9780203841624

[B42] Raykov T. Marcoulides G. A. (2012). Basic Statistics: An introduction with R. Lanham, MD: Rowman & Littlefield Publishers.

[B43] Raykov T. Marcoulides G. A. Gabler S. (2017). Improved estimation of maximal reliability for unidimensional multicomponent measuring instruments in repeated measure studies. Struct. Equ. Model. 24, 755–767. 10.1080/10705511.2016.1183491

[B44] Rutten E. A. Schuengel C. Dirks E. Stams G. J. J. M. Biesta G. J. J. Hoeksma J. B. (2011). Predictors of antisocial and prosocial behavior in an adolescent sports context. Soc. Dev. 20, 294–315. 10.1111/j.1467-9507.2010.00598.x

[B45] Schwarzer R. Jerusalem M. (1995). “Generalized self-efficacy scale,” in Measures in Health Psychology: A User's Portfolio. Causal and Control Beliefs, eds J. Weinman, S. Wright, and M. Johnston (Windsor, ON; England: NFER-NELSON), 35–37.

[B46] Sheard M. Golby J. van Wersch A. (2009). Progress toward construct validation of the sports mental toughness questionnaire (SMTQ). Eur. J. Psychol. Assess. 25, 186–193. 10.1027/1015-5759.25.3.186

[B47] Showers C. J. Ditzfeld C. P. Zeigler-Hill V. (2015). Self-concept structure and the quality of self-knowledge. J. Pers. 83, 535–551. 10.1111/jopy.12130 25180616 PMC4346517

[B48] Slimani M. Bragazzi N. L. Tod D. Dellal A. Hue O. Cheour F.. (2016). Do cognitive training strategies improve motor and positive psychological skills development in soccer players? Insights from a systematic review. J. Sports Sci. 34, 2338–2349. 10.1080/02640414.2016.1254809 27842463

[B49] Stanger N. Kavussanu M. Boardley I. D. Ring C. (2013). The influence of moral disengagement and negative emotion on antisocial sport behavior. Sport Exerc. Perform. Psychol. 2:117. 10.1037/a0030585

[B50] Stupuris T. Šukys S. Tilindiene I. (2013). Relationship between adolescent athletes' values and behavior in sport and perceived coach's character development competency. Balt. J. Sport Health Sci. 4, 37–45. 10.33607/bjshs.v4i91.178

[B51] Šukys S. Mankute G. (2012). Manifestation of prosocial and antisocial behaviour in a youth girls basketball match. Balt. J. Sport Health Sci. 4, 72–79. 10.33607/bjshs.v4i87.260

[B52] Vaughan R. Carter G. L. Cockroft D. Maggiorini L. (2018). Harder, better, faster, stronger? Mental toughness, the dark triad and physical activity. Pers. Individ. Dif. 131, 206–211. 10.1016/j.paid.2018.05.002

[B53] Vealey R. S. Chase M. A. (2008). “Self-confidence in sport,” in Advances in Sport Psychology, ed T. S. Horn (Champaign, IL: Human Kinetics), 66–97.

[B54] Vealey R. S. Knight B. (2002). “*Multidimensional sport-confidence: a conceptual and psychometric extension*,” in Paper presented at the Association for the Advancement of Applied Sport Psychology Conference (Tucson, AZ).

[B55] Vella S. (2019). Mental health and organized youth sport. Kinesiol. Rev. 8, 229–236. 10.1123/kr.2019-0025

[B56] Weiss M. (2013). Back to the future: research trends in youth motivation and physical activity. Pediatr. Exerc. Sci. 25, 561–572. 10.1123/pes.25.4.561 24214439

[B57] Zeiger J. S. Zeiger R. S. (2018). Mental toughness latent profiles in endurance athletes. PLoS ONE 13, e0193071–e0193071. 10.1371/journal.pone.0193071 29474398 PMC5825049

